# Daily Activity and Nest Occupation Patterns of Fox Squirrels (*Sciurus niger*) throughout the Year

**DOI:** 10.1371/journal.pone.0151249

**Published:** 2016-03-10

**Authors:** Thomas Wassmer, Roberto Refinetti

**Affiliations:** 1 Biology Department, Siena Heights University, Adrian, Michigan, United States of America; 2 Department of Psychology, Boise State University, Boise, Idaho, United States of America; University of Ferrara, ITALY

## Abstract

The authors investigated the general activity and nest occupation patterns of fox squirrels in a natural setting using temperature-sensitive data loggers that measure activity as changes in the microenvironment of the animal. Data were obtained from 25 distinct preparations, upon 14 unique squirrels, totaling 1385 recording days. The animals were clearly diurnal, with a predominantly unimodal activity pattern, although individual squirrels occasionally exhibited bimodal patterns, particularly in the spring and summer. Even during the short days of winter (9 hours of light), the squirrels typically left the nest after dawn and returned before dusk, spending only about 7 hours out of the nest each day. Although the duration of the daily active phase did not change with the seasons, the squirrels exited the nest earlier in the day when the days became longer in the summer and exited the nest later in the day when the days became shorter in the winter, thus tracking dawn along the seasons. During the few hours spent outside the nest each day, fox squirrels seemed to spend most of the time sitting or lying. These findings suggest that fox squirrels may have adopted a slow life history strategy that involves long periods of rest on trees and short periods of ground activity each day.

## Introduction

The level and pattern of an animal’s activity over the cycle of day and night, and over the time course of the seasons, are of profound importance for reproductive success [1–3] and survival [4–6]. The need to forage efficiently while being in danger of being injured or killed leads to a trade-off between starvation and predation risk that will affect the proportion of time per day to be active and foraging as well as the daily timing and recurrence of these processes [7–9]. Studies on both aspects of activity are, therefore, of utmost importance to understand the biology of a species.

The daily distribution of locomotor activity has been studied in detail in numerous species of vertebrates and invertebrates [10–12]. Among squirrels (family Sciuridae), many field and laboratory studies have examined daily and circadian rhythmicity at the individual level in various species of ground squirrels [13–18], chipmunks [19, 20], and marmots [21, 22]. The investigation of daily rhythmicity in arboreal squirrels has been limited, however. Several studies have reported the pooled and averaged activity patterns of groups of tree squirrels [23–27], but analysis of individual behavior has been limited to three laboratory studies—one on the American red squirrel, *Tamiasciurus hudsonicus* [28], and two on the southern flying squirrel, *Glaucomys volans* [29, 30]—and two field studies—one on the eastern gray squirrel, *Sciurus carolinensis* [31], and one on the fox squirrel, *Sciurus niger* [32]. Further studies on the circadian rhythmicity of tree squirrels are needed for completeness of the natural history record, especially because “tree squirrels” (sub family Sciurinae without genus *Sciurillus* but including flying squirrels, Pteromyinae) are phylogenetically distinct from “ground squirrels” (tribes Xerini, Marmotini, Protoxerini, and Funambulini) and separated from each other by at least 30 million years of divergent evolution into very different life styles and life strategies [33, 34].

To expand the knowledge about daily rhythmicity of activity in arboreal squirrels, we conducted an investigation of general activity and nest occupation patterns of fox squirrels (*Sciurus niger*) in a natural setting. The fox squirrel is the largest species of tree squirrel native to North America, measuring 45–70 cm (tail 20–30 cm) in length and weighing 500–1400 g [35–37]. The species shows no sexual dimorphism, and individuals may live up to 13 years in captivity. The natural range of the fox squirrel is the eastern half of the United States, excluding New England. The goals of the present study were to characterize the daily rest-activity cycle of individual fox squirrels, including the use of tree nests, to identify inter-individual differences in the activity pattern, and to investigate changes in the activity cycle along the seasons of the year.

## Materials and Methods

### Location

The study was conducted on an 80-ha area encompassing the campus of Siena Heights University and the adjacent Motherhouse campus of the Adrian Dominican Sisters, in rural Adrian, Lenawee County, in southeastern Michigan, USA (41°54’ N, 84°01’ W, 240 m elevation). Long-term climate data for Adrian are shown in Table 1. Weather conditions during the study were recorded by the Siena Heights University weather station and uploaded to Weather Underground (http://www.wunderground.com/personal-weather-station/dashboard?ID=KMIADRIA4).

**Table 1:** Long-term climate data for Adrian, Michigan.

Winter average low temperature	-8°C
Winter average high temperature	0°C
Summer average low temperature	15°C
Summer average high temperature	29°C
Annual average precipitation	863 mm

### Animals

Fox squirrels were caught with double-door Tomahawk Deluxe Transfer traps (Tomahawk Live Traps, Hazelhurst, WI) permanently attached to the trunk of four tall trees (one silver maple, *Acer saccharinum*, two catalpas, *Catalpa speciosa*, and one Norwegian spruce, *Picea abies*) on custom-made platforms [38]. Squirrels were weighed with Pesola spring scales and ear-tagged with monel type 5 tags (National Band and Tag Company, Newport, KY). Age was categorized as juvenile, sub adult, and adult according to weight, fur characteristics, and by visual examination of external reproductive organs. Sex of adult and sub-adult squirrels was also determined by visual examination of the external reproductive organs [39, 40]. All work was conducted with approval of the Institutional Animal Care and Use Committee of Siena Heights University and followed appropriate guidelines as outlined by Sikes and colleagues [41].

### Data Collection

Squirrels were equipped with a temperature-sensitive data logger collar. The monitoring of collar temperature is particularly valuable in providing an index of location (in-nest vs. out-of-nest), as the temperature of the collar rapidly rises to body temperature when the animal enters the thermally-insulated nest and drops rapidly when the animal leaves the nest [20, 42, 43]. Because of changes in ambient temperature related to microclimate variations as the animal moves around, the monitoring of collar temperature additionally provides an index of locomotor activity comparable to that obtained by actigraphy or telemetry.

The data logger collars that we used incorporated temperature-sensitive iButtons (DS1922L, Maxim Integrated Products, Inc., San Jose, CA). The iButtons measure 17 mm in diameter and are 6.5 mm thick, weighing 2.5 grams. They were programmed to record one temperature reading every 1, 5, 10 or 30 minutes with a thermal accuracy of 0.5 or 0.0625°C. This allowed data to be recorded for a minimum of 5 days (at 1 min intervals with 0.5°C temperature resolution), 28.5 days (at 5 min intervals with 0.5°C resolution, or at 10 min intervals with 0.0625°C resolution) and a maximum of 5.7 months (at 30 min intervals with 0.5°C resolution). To prevent water penetration, iButtons were first waxed (Paramat Extra, Electron Microscopy Sciences, Hatfield, PA) and then encased into acrylic dental resin (Jet Tray, Lang Dental Manufacturing Co., Wheeling, IL). The polymerization of the resin was used to attach the casing to a parallel-entry cable tie. For comfort, the cable tie was padded with heat shrink. The total package weight was between 8 and 12 grams and did not exceed 2% of the animal’s body mass. Collars were attached with the iButton logger above the throat of the fox squirrels. Due to their weight, data loggers will typically stay below the chin of the animals and expose the sensors to the air temperature in active animals but approach body temperature when fox squirrels are curled up in rest.

Only squirrels that returned repeatedly to the traps were chosen for the study, as retrieval of the data loggers was essential for data processing. In the interval from October 2011 to May 2015, we obtained data from 25 distinct preparations, upon 14 unique squirrels, totaling 1385 recording days.

To verify that changes in T_c_ were indeed related to changes in behavior and location, we conducted short-term visual observations of individual squirrels. For these observations, iButtons were programmed to record T_c_ with 1-min resolution. Behavioral observations were conducted by an investigator experienced in the study of sciurid behavior and with the assistance of the iPhone application Animal Behaviour Pro (School of Anthropology and Conservation, University of Kent, Canterbury, UK).

### Data Analysis

Presence of statistically-significant 24-hour rhythmicity was determined by three methods: chi-square periodogram [44], Lomb-Scargle periodogram [45], and cosinor rhythmometry [46]. Rhythm robustness, which is an index of day-to-day consistency of the rhythmic pattern, was computed as the percentage of total variance accounted for by the cosine fit [47]. Standard statistical tests were used for comparisons of group means [48].

Exact times of entry into and exit from the nest were often easily identified by visual inspection of collar temperature (T_c_) records. To avoid potential subjectivity bias, however, the time series were analyzed by a custom-made computer program. The computer algorithm defined a nest entry as the data point when the value of T_c_ met two requirements: 1) the current T_c_ was higher than the preceding T_c_, and 2) the current T_c_ was higher than or equal to the mean nightly T_c_ of the individual squirrel. Although this simple algorithm worked well for data sets with a temperature gradient of more than 10°C between T_c_ and T_a_, additional specifications had to be included to ensure successful data analysis during warmer periods that resulted in more noisy data sets. For the second criterion, the current T_c_ had to exceed the mean nightly T_c_ minus one standard deviation of the mean, and this had to be true also for the two T_c_ values following the current T_c_ value. Analogous requirements were used to calculate the times of exit from the nest. Days in which the program failed to identify at least one exit from and one return to the nest were omitted from further analysis. These cases accounted for less than 2% of the total days available for analysis.

The durations of days and nights were calculated for each day using the times of civil dawn and civil dusk as computed with basis on the local latitude, longitude, date, and geopolitical time zone. Changes in official time due to the beginning and end of daylight-saving time were ignored, so not to distort the time series. The correctness of twilight computations was verified against values posted by the Astronomical Applications Department of the U.S. Naval Observatory (http://aa.usno.navy.mil/data/docs/RS_OneYear.php).

Inter-individual differences in the temporal pattern of T_c_ oscillation were evaluated by visual observation of the time series and by comparison of the coefficients of variation (standard deviation divided by the mean) of the various parameters that were computed as described above.

## Results

**Fig 1:**
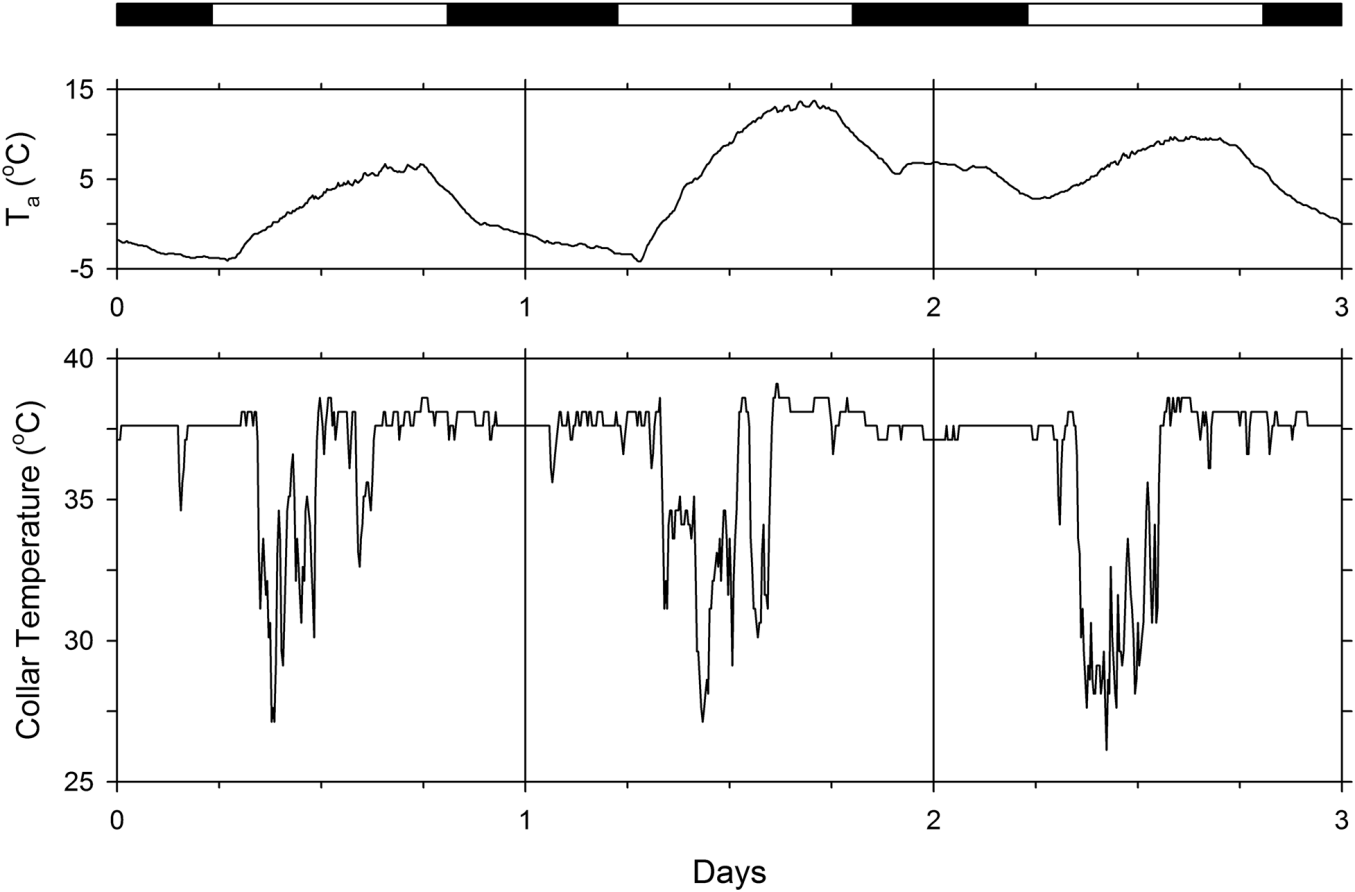
Three-day segment of the records of collar temperature of a male fox squirrel in the spring. The horizontal bar above the graph denotes the duration of the natural light-dark cycle (with white denoting light and black denoting darkness). T_a_ is air temperature as recorded by a weather station located a few hundred meters from the site.

Most squirrels on most days exhibited a clear daily rhythm of collar temperature (T_c_), as exemplified in Fig 1. On the first day, T_c_ was high and relatively stable while the animal rested in its nest at night, then fell noticeably a few hours after sunrise as the squirrel left the nest. The squirrel returned to the nest for a few hours around noon and then made a brief excursion out of the nest before retiring for the night. Similar patterns are seen on the second and third days of this three-day segment of a 38-day recording session. On all three days, rapid oscillations in T_c_ are seen when the animal is outside the nest, which indicates movement across or exposure to various microclimates. Notice that, although ambient temperature oscillated daily, the daily variations in collar temperature were independent from variations in ambient temperature. When analyzed in blocks of at least 10 consecutive days, data from all 25 recording sessions with 14 squirrels exhibited significant 24-hour rhythmicity (*p* < 0.0001), as determined by the three distinct procedures (chi-square periodogram, Lomb-Scargle periodogram, and cosinor rhythmometry).

**Fig 2:**
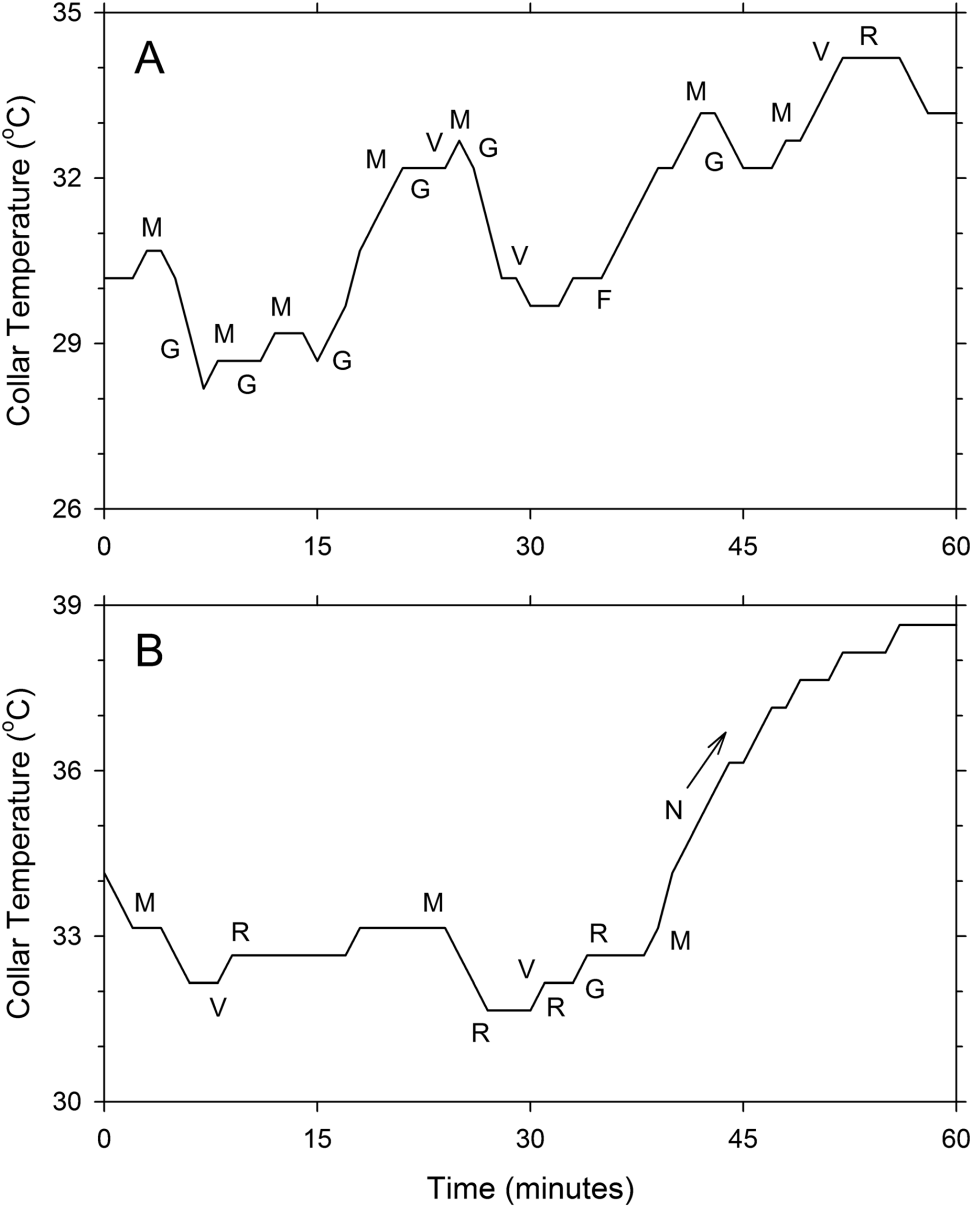
Relationship between collar temperature and animal behavior in one-hour segments of the records of a female (A) and a male (B) fox squirrel. Ambient temperature was 15°C in both cases. Abbreviations: F, feeding; M, moving; N, inside the nest; G, grooming; R, resting; and V, vigilance.

Two examples of visual observation records of individual squirrels are shown in Fig 2. It can be seen that changes in T_c_ are mostly associated with moving and grooming, whereas stable T_c_ is associated with resting and vigilance. One of the squirrels (Panel A) remained outside the nest for the one-hour observation period, and Tc never exceeded 35°C. The other squirrel (Panel B) entered the nest after approximately 40 minutes, and a steep elevation in T_c_ can be seen afterwards, reaching past 38°C after about 15 minutes.

**Fig 3:**
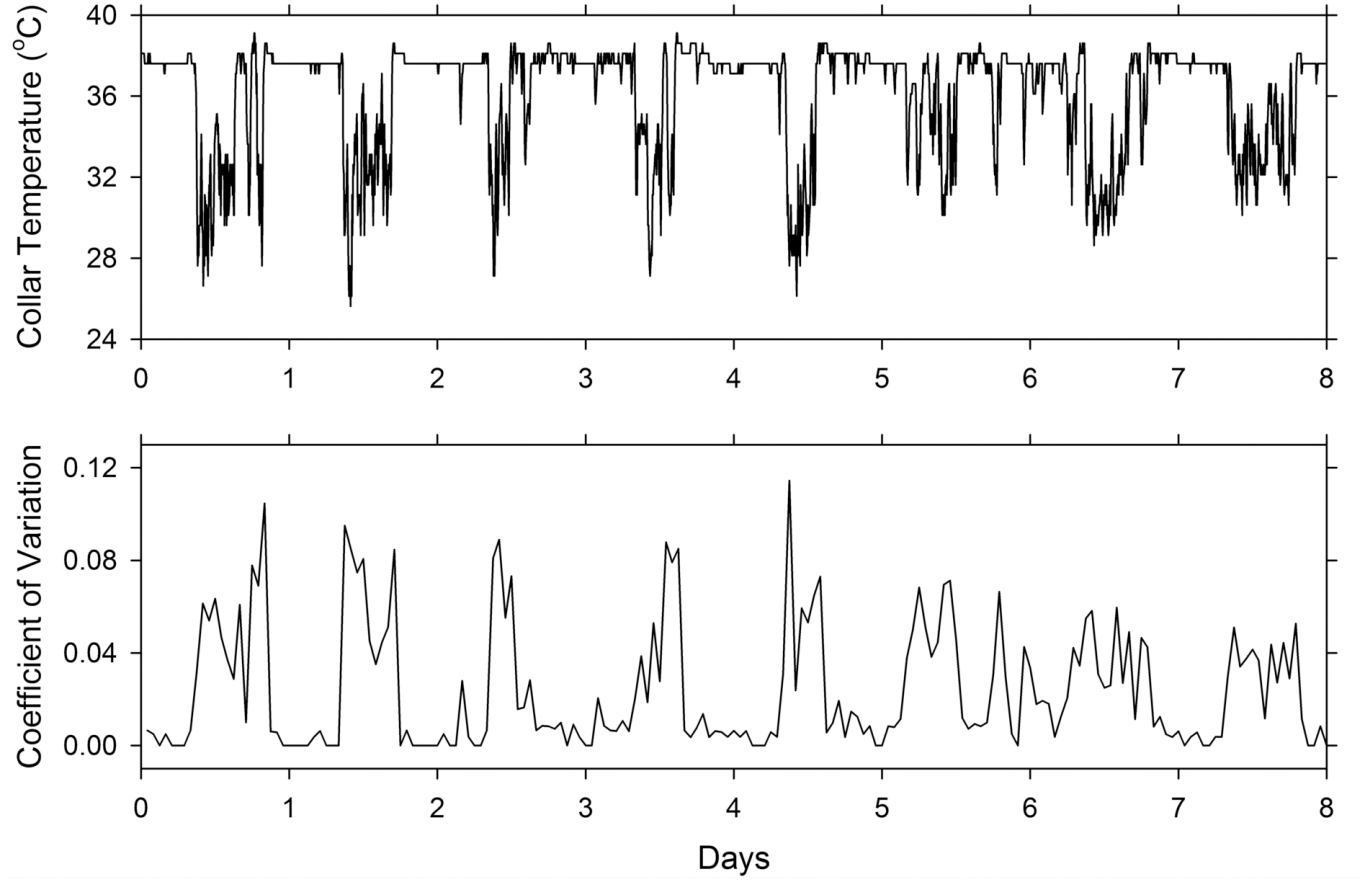
Eight-day segment of the records of collar temperature (top) and its corresponding activity record as computed by the hourly coefficient of variation of the temperature measurements (bottom).

To further validate the use of collar temperature for the measurement of activity, we used a procedure equivalent to that used for the monitoring of activity by radio telemetry (that is, variation in signal strength). We took T_c_ records with 5-min resolution (Fig 3, top) and calculated the variability of this signal over an hour (12 data points). We expressed this variability as a coefficient of variability (standard deviation divided by the mean) with 1-hour resolution (Fig 3, bottom). It can be seen that, except for the loss of resolution, the waveform of the coefficient of variability is a very close mirror image of T_c_. Thus, the original T_c_ record is a reliable measure of activity (and is preferable to the derived variable because of its greater temporal resolution).

**Fig 4:**
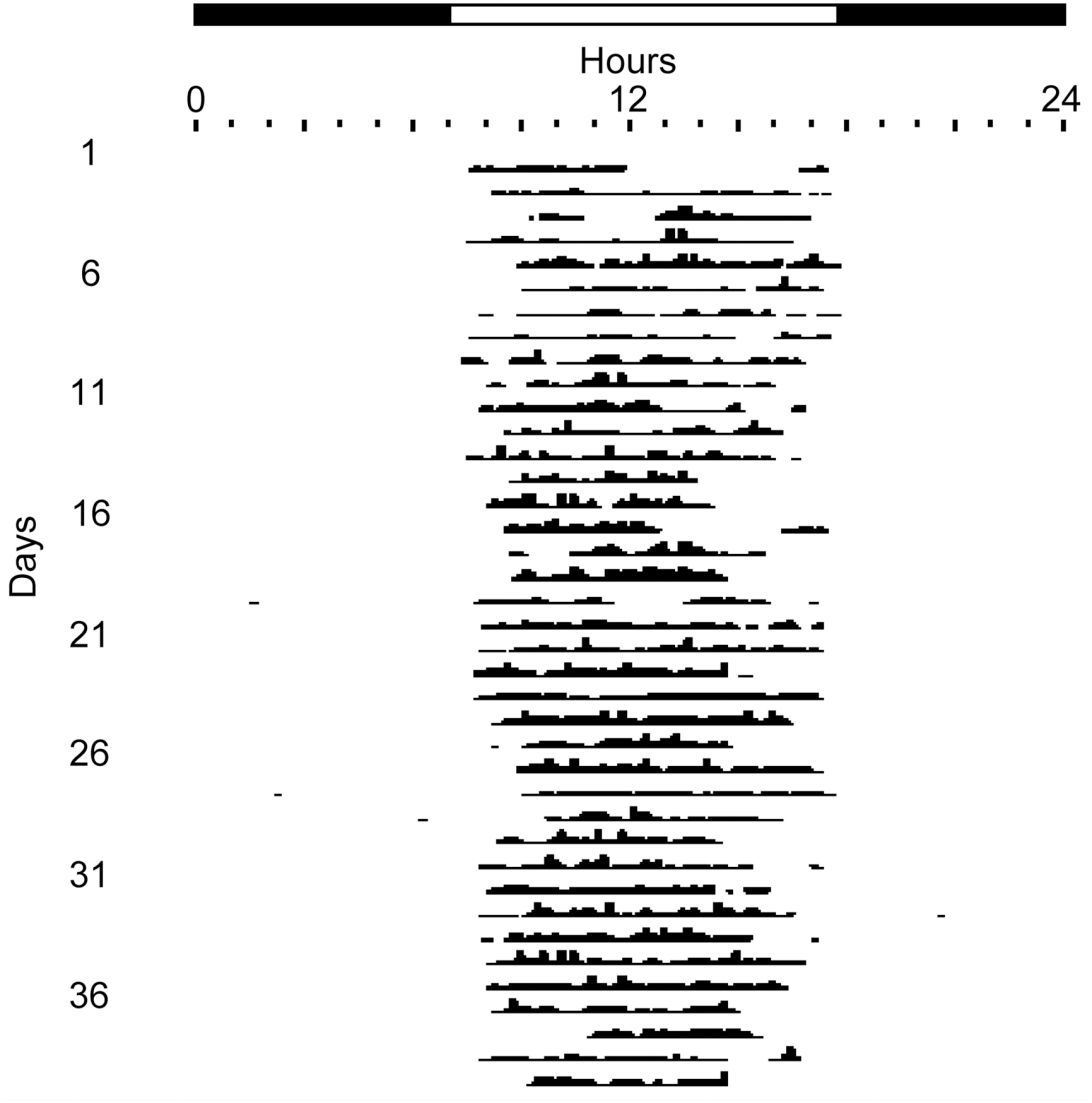
Actogram-style plot of 38 consecutive days of the records of collar temperature of a female fox squirrel in the autumn. Each line corresponds to a day, and consecutive days are plotted below each other. Each day, temperatures below the daily mean are plotted proportionally to the difference from the mean. The horizontal bar above the graph denotes the duration of the natural light-dark cycle.

The records of another individual squirrel are shown in actogram format in Fig 4. “Onsets” and “offsets” of activity show day-to-day variability but are consistently restricted to the interval between dawn and dusk. The distribution of activity each day is predominantly unimodal (or flat) rather than bimodal.

**Fig 5:**
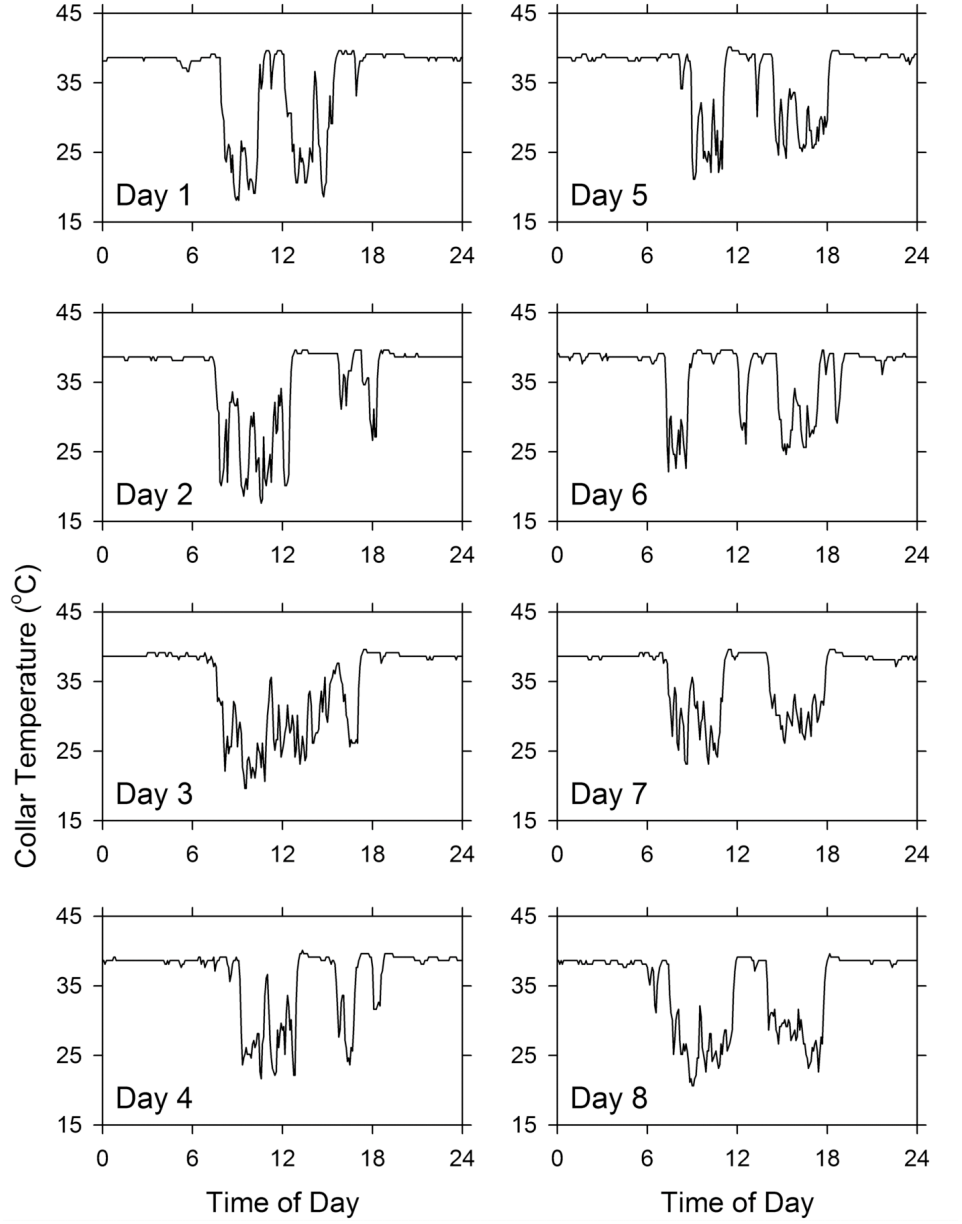
Records of collar temperature of a female fox squirrel studied during the spring. Each panel shows data for one day, as indicated. The data were collected in 5-min intervals.

**Fig 6:**
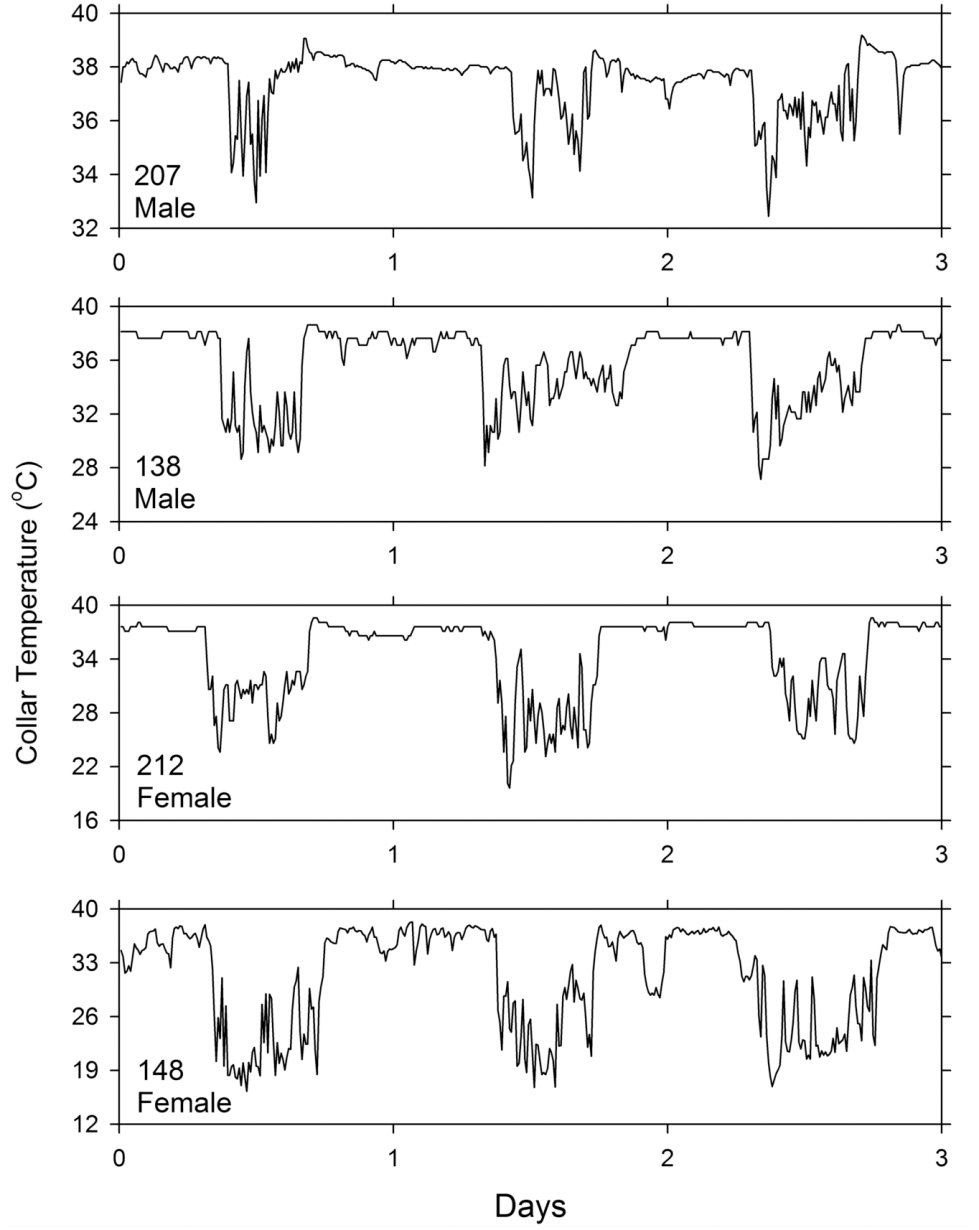
Records of collar temperature of two male and two female fox squirrels. Each panel shows data for three consecutive days. The data were collected in 10-min intervals.

Records from another squirrel are shown as raw time series in Fig 5. Although a bimodal pattern (with deflections in the morning and in the afternoon) can be clearly seen on some days (such as Days 1, 5, 7, and 8), the pattern is variable from day to day and is absent on other days. This animal’s activity pattern is representative of the activity patterns of the other squirrels. Although some squirrels exhibited bimodal activity patterns on some days (with noticeable T_c_ troughs at dawn and dusk), the pattern was not constant and vanished when averaged over several days. Three-day segments of the raw data of two male and two female squirrels are shown in Fig 6.

**Fig 7:**
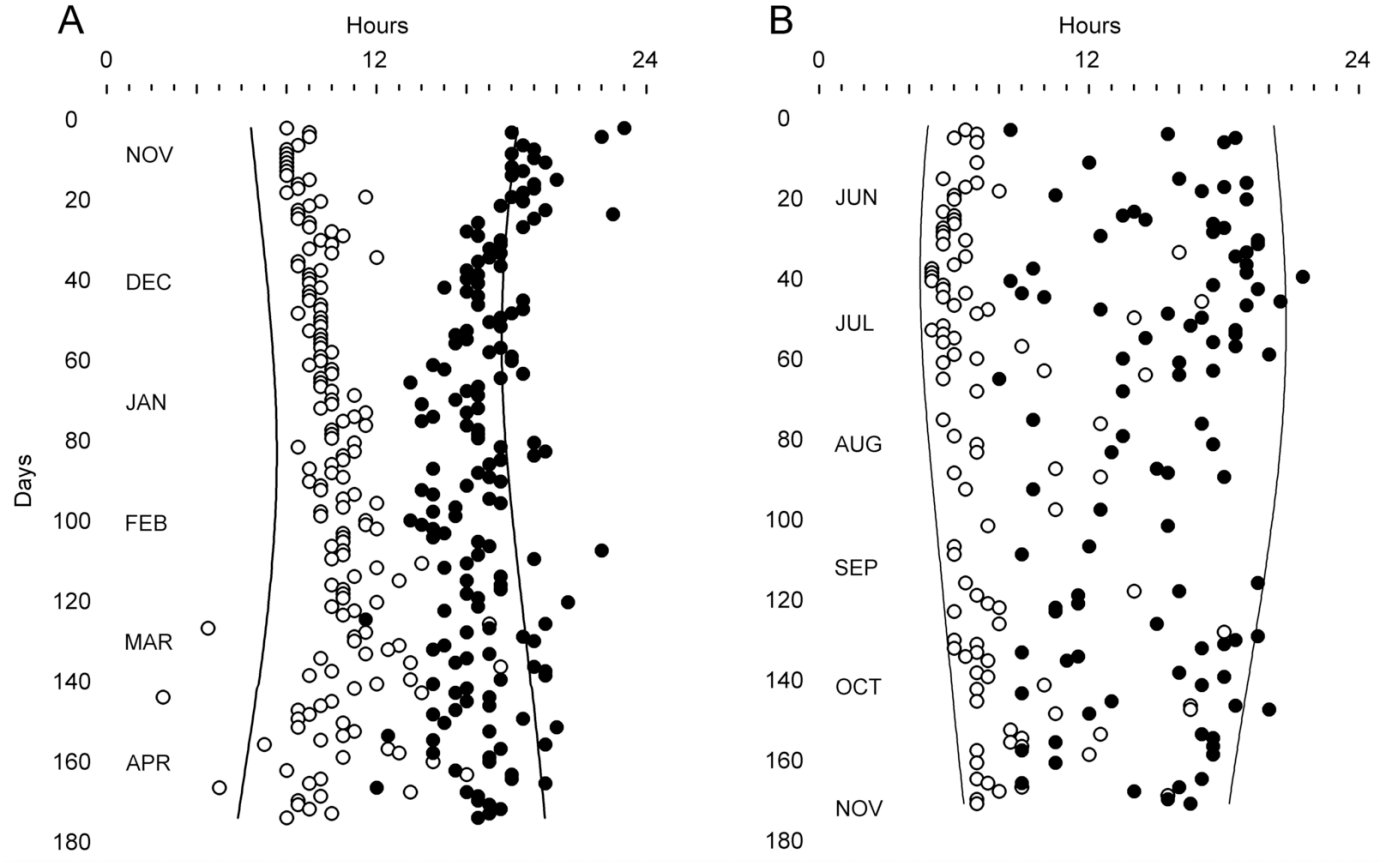
Times of first daily exit from the nest (open circles) and last return to the nest (closed circles) of two fox squirrels (A and B) during six consecutive months in the field. The times of civil twilights are indicated by the continuous vertical lines.

Daily patterns differed not only from squirrel to squirrel but also from season to season. As exemplified for two squirrels in Fig 7, the times of exit from the nest and re-entry into the nest varied slightly but noticeably as the days became longer in the summer and shorter in the winter. The animal whose records are shown in Panel B exhibited more day-to-day variability in nest-exit times and, particularly, in nest-return times than the animal whose records are shown in Panel A. Conversely, the squirrel whose records are shown in Panel A had more nocturnal returns to the nest than the squirrel whose records are shown in Panel B despite displaying smaller inter-day variability in return times.

Mean results for all animals are summarized in Table 2. Rhythm robustness, which is an index of day-to-day consistency of the temporal pattern, was moderate (grand mean: 25%) and did not vary with the seasons (*F*_3, 33_ = 0.323, *p* = 0.809). The time of the first daily exit from the nest was significantly affected by the seasons when expressed in local clock time (*F*_3, 33_ = 8.714, *p* < 0.001), with the nest-exit time being 2.1 hours earlier in the summer than in the winter. However, because days are longer and start earlier in the summer, it is necessary to consider also the time of the first daily exit when expressed as number of hours after dawn. The squirrels exited the nest between 1.5 and 2.2 hours after dawn, without statistically significant seasonal differences (*F*_3, 33_ = 0.323, *p* = 0.809), which indicates that they tracked dawn as the days became shorter and longer. Interestingly, the time spent out of the nest was not affected by the seasons (*F*_3, 33_ = 0.973, *p* = 0.417), with approximately 7 hours being spent out of the nest each day regardless of season. It should be noted that, at this location, day length varies from 9 hours in the winter to 15 hours in the summer, so that even in the winter the animals did not take advantage of all available hours of sunlight for foraging (or for performing other out-of-nest activities).

**Table 2:** Parameters of the activity rhythm over the seasons. *S*.*E*.*M*.: standard error of the mean, *n*: sample size (animals).

		Rhythm robustness (%)	Time of first exit (clock time)	Time of first exit (hours after dawn)	Time spent out of nest (hours)	Number of exits per day
**Winter**	***Mean***	27	9:27	1.60	6.7	1.13
	***S*.*E*.*M*.**	5	0:14	0.24	0.3	0.03
	***n***	8	8	8	8	8
**Spring**	***Mean***	24	7:51	1.90	7.7	1.40
	***S*.*E*.*M*.**	2	0:22	0.36	0.6	0.05
	***n***	10	10	10	10	10
**Summer**	***Mean***	22	7:23	2.18	7.1	1.37
	***S*.*E*.*M*.**	3	0:21	0.36	0.8	0.02
	***n***	8	8	8	8	8
**Autumn**	***Mean***	26	8:47	1.53	7.8	1.13
	***S*.*E*.*M*.**	5	0:13	0.22	0.40	0.03
	***n***	11	11	11	11	11

Despite spending the same number of hours out of the nest throughout the year, the squirrels made slightly but significantly more excursions out of the nest in the spring and summer (1.4 excursions per day) than in the autumn and winter (1.1 excursions per day) (*F*_3, 33_ = 14.610, *p* < 0.001). This means that there were a few more days with bimodal activity patterns in the spring and summer than in the autumn and winter, although the preponderant pattern was unimodal throughout the year.

By computing the coefficient of variation (standard deviation divided by the mean) of parameters in Table 2, it can be noted that there is considerable inter-individual variability in the number of exits from the nest each day (CV = 0.79), not as much in rhythm robustness (CV = 0.46), and even less in time spent out of the nest each day (CV = 0.21). There is more inter-individual variability in these behavioral variables than in a structural variable such as adult body mass (CV = 0.11).

Although we did not collect quantitative data on activities performed by all fox squirrels outside the nest, visual observations of several squirrels over several hours indicated that the animals were sitting or lying quietly, apparently resting, during 45–67% of the time spent outside the nest.

## Discussion

Fox squirrels free-ranging in an arboreal suburban environment exhibited robust daily rhythmicity of locomotor activity, as gauged by changes in the temperature of their microenvironments. Rhythm robustness averaged 25%, which is lower than the robustness of the activity rhythms of laboratory mice and hamsters recorded under controlled laboratory conditions but is comparable to the robustness of the rhythms of laboratory rats and gerbils also recorded under controlled laboratory conditions [49]. Rhythm robustness refers to the strength and regularity of the daily oscillations, so that the finding of rhythm robustness comparable to that of laboratory rats and gerbils means that fox squirrels in the field organize their activity with regularity similar to that of rats and gerbils in the laboratory. Controlled studies with fox squirrels would be needed to determine how much of the regularity is due to the precision of the internal clock and how much is the result of environmental factors.

We found the daily activity pattern of the fox squirrel to be predominantly unimodal, without clusters of activity at dawn and dusk, as the squirrels exited their nests an average of 1.3 times each day. Although using a lower temporal resolution of one hour, Adams [32] also observed a unimodal activity pattern in individual fox squirrels. In our study, the activity pattern changed only slightly with the seasons, as the squirrels made a few more excursions out of the nest in the spring and summer (1.4 excursions per day) than in the autumn and winter (1.1 excursions per day). This is in contrast with previous descriptions of a strong seasonal change from a unimodal pattern in the winter to a bimodal pattern in the summer in the big cypress fox squirrel, *Sciurus niger avicennia* [23, 24], Mexican fox squirrel, *Sciurus nayaritensis chiricahuae* [25] and Eurasian red squirrel, *Sciurus vulgaris* [27]. The activity patterns of our fox squirrels showed a high degree of variability between different individuals during the same sampling period and to a lesser extent between the recordings of the same individual in different seasons. Individual patterns changed from day to day, possibly because of changing weather conditions and perhaps also because of encounters with predators or other events. Nonetheless, the fact that our fox squirrels made significantly (though moderately) more excursions out of the nest in the spring and summer is the reflection of a higher incidence of bimodal activity patterns during these two seasons than during the autumn and winter.

Although fox squirrels were clearly diurnal, as previously noted by others [28, 32, 50], they occasionally left the nest at night (as determined by the T_c_ records, e.g. Fig 6 bottom panel). Even during the short days of winter (9 hours of light), the squirrels typically left the nest after dawn and returned before dusk, spending only about 7 hours out of the nest each day. Restriction of activity to a short window during daylight has been previously described in detail for European ground squirrels [51] and European hamsters [52]. In agreement with Koprowski and Corse’s findings in Mexican fox squirrels [25], we observed that fox squirrels spent more than half of their out-of-nest time resting rather than feeding or moving.

Expansion and contraction of the daily activity interval associated with the change of the seasons have been documented in various species [53–57]. In this study on fox squirrels, the time spent out of the nest each day was not significantly affected by the seasons, but the time of the first daily exit did vary with the seasons. The squirrels exited the nest earlier in the day when the days became longer in the summer and exited the nest later in the day when the days became shorter in the winter, thus tracking dawn along the seasons. Tracking dawn is a strategy expected of animals with free-running periods longer than 24 hours, as these animals need to be exposed to light during the phase-advance region of their photic phase-response curve [58]. We do not know the free-running period of the fox squirrel, but it is likely that it exceeds 24 hours because the free-running periods of various other diurnal squirrels exceed 24 hours [12].

It is interesting to compare our results on fox squirrels with the results obtained by Tester on gray squirrels [31], as these two species are the two main, co-existing squirrel species in the eastern United States. Because of differences in methods between the two studies, we will limit the comparison to the duration of the daily period of activity. Even during the long days of summer, Tester found that gray squirrels initiate activity before dawn and terminate after dusk, whereas our results indicate that fox squirrels restrict their activity to a 7-hour window during sunlight. Interestingly, the shortest daily activity time observed by Tester was 8 hours in the winter, which is one hour longer than the constant 7-hour window of the fox squirrel. The short duration of activity in the fox squirrel, together with the high percentage of resting when outside of the nest, may be an indication of a slow life history strategy [59, 60]. It is possible that fox squirrels minimize predator pressure by spending almost their entire life on trees [61] and preserve energy by resting as much as possible. This might be a major factor (in addition to scatter hoarding) that allows them to stay normothermic throughout winter without any substantial increase in plumage or nest insulation. However, further studies comparing all aspects of the life history and lifestyle of fox squirrels are necessary to substantiate this inference.

One limitation of this study is that the selection of research subjects was not fully randomized. Because we could only analyze the data from squirrels that were recaptured, we cannot exclude the possibility that our results are limited to squirrels with small home ranges. Fox squirrel home ranges vary from 1 to 40 ha [35], but we did not investigate the exact home range of the squirrels living in our 80-ha study area.
